# Biosynthesis and Signal Transduction of ABA, JA, and BRs in Response to Drought Stress of Kentucky Bluegrass

**DOI:** 10.3390/ijms20061289

**Published:** 2019-03-14

**Authors:** Yajun Chen, Yang Chen, Zhenjie Shi, Yifeng Jin, Huashan Sun, Fuchun Xie, Lu Zhang

**Affiliations:** 1 College of Horticulture and Landscape Architecture, Northeast Agricultural University, Harbin 150030, China; chenyajun622@163.com (Y.C.); chenyang8368215@hotmail.com (Y.C.); shizj95516@163.com (Z.S.); huashan9303@163.com (H.S.); xfc204309@163.com (F.X.); 2 College of Life Science, Agriculture and Forestry, Qiqihar University, Qiqihar 161006, China; Jinyifeng8368215@163.com

**Keywords:** differentially expressed genes, drought stress, hormone signaling, Kentucky bluegrass, transcription factors, RNA-Seq

## Abstract

Kentucky bluegrass (KB, *Poa pratensis*) is one of the most widely used cool-season turfgrass species, but it is sensitive to drought stress. Molecular studies in KB are hindered by its large and complex genome structure. In this study, a comparative transcriptomic study was conducted between a short and long period of water deficiency. Three transcriptome libraries were constructed and then sequenced by using leaf RNA samples of plants at 0, 2, and 16 h after PEG6000 treatment. A total of 199,083 differentially expressed genes (DEGs) were found. The Kyoto Encyclopedia of Genes and Genomes (KEGG) annotation revealed that DEGs were enriched in “Plant hormone signal transduction” and “MAPK signaling pathway-Plant”. Some key up-regulated genes, including *PYL*, *JAZ*, and *BSK*, were involved in hormone signaling transduction of abscisic acid, jasmonic acid, and brassinosteroid and possibly these genes play important roles in coping with drought stress in KB. Furthermore, our results showed that the concentrations of ABA, JA and BR increased significantly with the extension of the drought period. The specific DEGs encoding functional proteins, kinase and transcription factors, could be valuable information for genetic manipulation to promote drought tolerance of KB in the future.

## 1. Introduction

Lawns are part of the green ecosystem in the urban area which provides many kinds of environmental functions such as CO_2_ fixation and emission of O_2_. Kentucky bluegrass (“KB”) (*Poa pratensis* L.) is an excellent cool-season turfgrass and is extensively used in public parks, golf courses and residential lawns. This species possesses highly ornamental value, but it is extremely sensitive to water scarcity [1]. This important grass species has a large and complex genome that hinders its molecular and genetic studies. In particular, KB has hexaploid, octoploid and heptaploid varieties with a DNA per cell value of 6–9 pg for most varieties, which is about 3–4 times larger than that of maize (*Zea mays*) (data source from http://data.kew.org/cvalues/; July 2013). In addition, KB exhibits both sexual fertilization and apomixis with respect to seed formation [2]. As for its sexual fertilization, KB is self-incompatible and therefore has a highly heterozygous genome background. So far, the genome sequence of this species has not been released. With the development of the next-generation sequencing technology, *de novo* transcriptome analysis without a reference genome is considered an alternative way to identify putative genes that are related to many important physiological processes.

Global climate change has increased the occurrence of drought globally, due to which new challenges have been raised for turfgrass management. Drought has become a pivotal factor negatively affecting turfgrass growth and quality [3]. Drought stress significantly impacts water status by reducing the relative water content in plants [4,5]. Plants evolved different strategies to cope with short or long durations of drought stress. For example, stomatal closure is an effective response of plants to reduce water loss during drought stress [6]. Prolonged drought stress causes osmotic and oxidative stresses, resulting in an increase in reactive oxygen species (ROS) and impairment of plant cells [7]. In our previous studies, we found that drought stress notably influenced agronomical, anatomical and physiological attributes of KB, such as relative water content (RWC), leaf crosscutting structure, and photosynthesis [8,9]. Drought stress also causes the accumulation of phytohormones such as abscisic acid (ABA), jasmonic acid (JA), and salicylic acid in KB [10]. Furthermore, drought stress remarkably increased the antioxidant enzyme activities of KB [1]. However, potential genes underlying such physiological changes have not yet been studied mainly due to the lack of genomic and bioinformatics resources of this specific grass species.

Physiological responses as well as their underlying molecular responses to drought stress change during the time course. Drought stress affects both physiological parameters and gene expression. For example, one study documented that the photosynthesis parameters (stomatal conductance, transpiration rate, and net photosynthetic rate) of rice seedlings decreased rapidly at 3 h and thereafter exhibited an increasing trend at 48 h under PEG-simulated drought stress [11]. One PEG-inducible gene (*OsASR5*), which is involved in stomatal closure, exhibited the highest and lowest expression level at 3 and 16 h, respectively, after PEG treatment in rice [12]. *Monodehydroascorbate reductase* and *glutathione reductase* were up-regulated by drought stress and remained at those levels even after recovery in KB [13]. Peanut (*Arachis hypogaea*) plants exposed to PEG stress during the fruiting stage resulted in ABA accumulation initially at 1 h, and then it achieved its relatively higher level after 16 h of stress [6]. However, it is still not clear whether phytohormone biosynthesis and signaling transduction can change dynamically in KB upon drought stress. Therefore, the potential difference between short- and long-term drought response should be taken into account to further understand the response to drought of KB.

In order to cope with abiotic stresses, plants exhibit comprehensive mechanisms such as signal transduction, metabolic activation, and gene expression regulation [14,15,16]. Transcriptome profiling has been used widely to identify drought-responsive genes and characterize molecular mechanisms [17]. Functions of stress responsive genes such as heat stress transcription factor (*HSF*), dehydration-responsive element binding proteins (*DREB*), serine/threonine protein kinase (*SnRK2*), lipid transfer protein (*LTP*), and *MYB* have been further verified and well characterized [18,19,20,21,22,23]. These genes are promising candidates for promoting drought tolerance. As the most popular technique in transcriptome profiling, RNA-Seq has been broadly used in turfgrass research [24]. The results derived from RNA-Seq showed that oxidative protection, proline biosynthesis, and lipid hydrolysis played key roles in response to heat stress in bentgrass (*Agrostis scabra*) [25]. It has been revealed that transcription factors, ion and water transport genes of KB played key roles during salt tolerance [26]. However, there is no report on transcriptomic analysis of KB in different drought periods using RNA-Seq.

In the present study, we identified drought stress responsive genes using RNA sequencing. The objectives of this study were: (1) to provide a bioinformatics resource for KB with a focus on drought-inducible genes; (2) to understand dynamic changes of key metabolic pathways during short-term drought periods with an emphasis on plant hormone signal transduction over time.

## 2. Results

### 2.1. Sequence Analysis and Transcript Assembly

A total of 380 million clean reads were identified after trimming adapters and filtering out low-quality reads (Supplementary Table S1). The number of clean reads obtained by each group accounted for more than 94.64% of the original reads, which indicated that the libraries worked well. The sequence length obtained by splicing was used to measure the splicing quality. After Trinity splicing, the total length of the transcript was 643,096 nt, including 96,544 scaffolds larger than 1000 bp. The average transcript length was 677.96 nt, N_50_ length was 747 bp, and N_90_ length was 374 bp (Supplementary Table S2) after local assembly with the unmapped ends to fill in the small gaps within the Scaffolds. The *de novo* assembly yielded 199,083 unigenes with an average length of 718.83 bp.

### 2.2. Sequence Annotation

A total of 199,083 unigenes were annotated, and many of them were in the TrEMBL (110,075 and 55.29%) and the NR (109,922 and 55.21%) database. In addition, 3120 unigenes (1.57%) were annotated to nine databases, and 122,431 unigenes (61.5%) obtained functional annotations in at least one database (Supplementary Table S3). In order to analyze the conservation of sequences, the unigenes annotated in the NR database of KB were compared with those of other species (Supplementary Figure S1). The top match was *Brachypodium distachyon* (27,695 and 25.14%), followed by *Aegilops tauschii* (18,969 and 17.22%), *Hordeum vulgare* subsp. *vulgare* (18,783 and 17.05%), *Triticum urartu* (10,617 and 9.64%). As expected, more than 86.88% of the sequences were highly homologous with the sequences of Gramineous plants.

**Figure 1:**
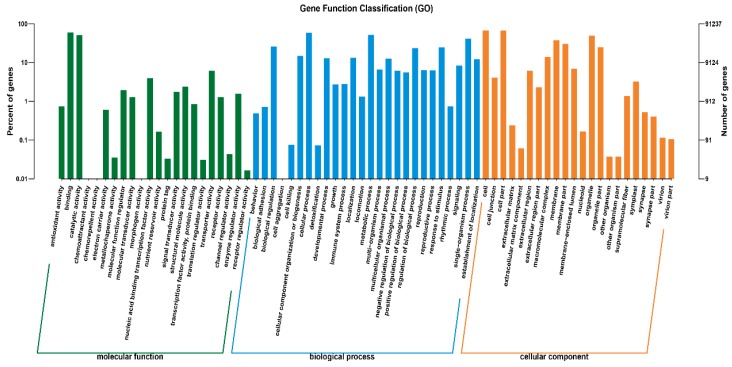
Bar plots of Gene Ontology (GO) classifications of the assembled sequences from the Kentucky Bluegrass transcriptome.

**Figure 2:**
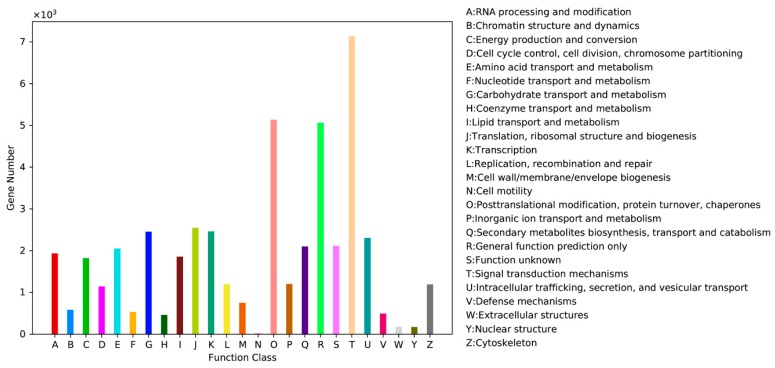
Histogram presentation of clusters of orthologous groups from the Kentucky Bluegrass transcriptome.

A total of 91,237 unigenes were annotated to the GO database, with three main ontologies: biological process, cellular component and molecular function. “Cellular process”, “Metabolic process” and “Single-organism process” were the most abundantly represented subcategories in the biological process, “cell”, “cell part” and “organelle” in cellular components categories (Figure 1). Within the molecular function, “Catalytic activity”, “Binding” and “Transporter activity” contained the largest number of genes (Figure 1). In total, 41,991 unigenes annotated by KOG were classified into 25 categories (Figure 2). The largest number of unigenes were classified as “Signal transduction mechanisms” (7130 and 15.19%), followed by “Posttranslational modification, protein turnover, chaperones” (5133 and 10.94%), and “General function prediction only” (5065 and 10.79%).

**Figure 3:**
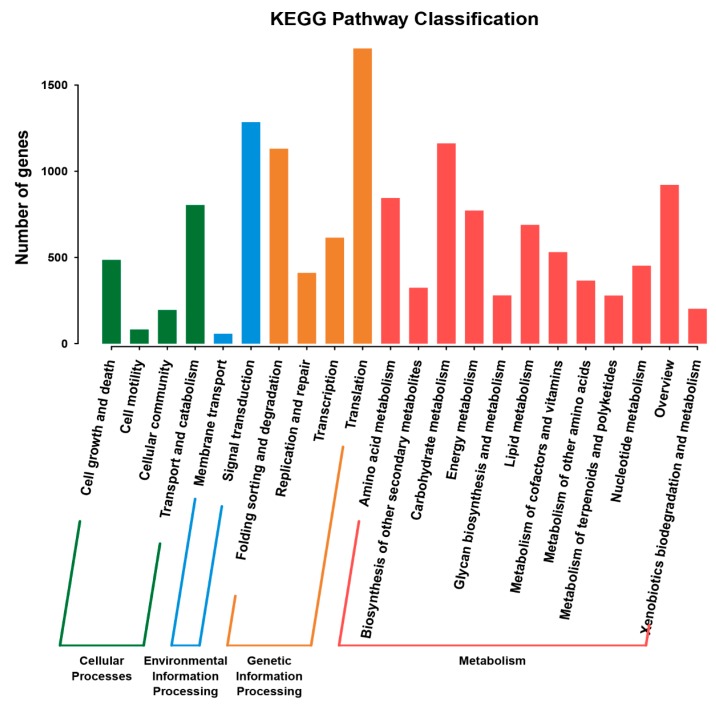
Plots showing categories of genes classified based on the Kyoto Encyclopedia of Genes and Genomes (KEGG) analysis.

KEGG is used for metabolic analysis and metabolic network research in organisms. In the present study, 13,601 unigenes were classified into 214 KEGG pathways. The pathways including “Cellular processes”, “Environmental information processing”, “Genetic information processing”, and “Metabolism”. The “Metabolism” pathway contained the largest number of unigenes (6823 and 50.17%), followed by “Genetic information processing” (3869 and 28.45%), and “Cellular processes” (1567 and 11.52%) (Figure 3).

### 2.3. DEGs under Drought Stress

The reliability of the libraries was evaluated using repeated correlation and cluster analysis of samples (Supplementary Figures S2 and S3). There were 15,536 up-regulated and 14,770 down-regulated genes in the 2 *vs* 0 h regime (Figure S4B). In the 16 vs 2 h regime, 16,420 up-regulated and 14,498 down-regulated genes were found. In 16 *vs* 0 h, 5110 up-regulated and 4030 down-regulated genes were identified (Supplementary Table S4). A Venn diagram analysis indicated that 24 genes were up-regulated while 36 genes were down-regulated continuously during the entire drought period (Figure S4A, Supplementary Table S4).

### 2.4. GO Analysis of DEGs

**Figure 4:**
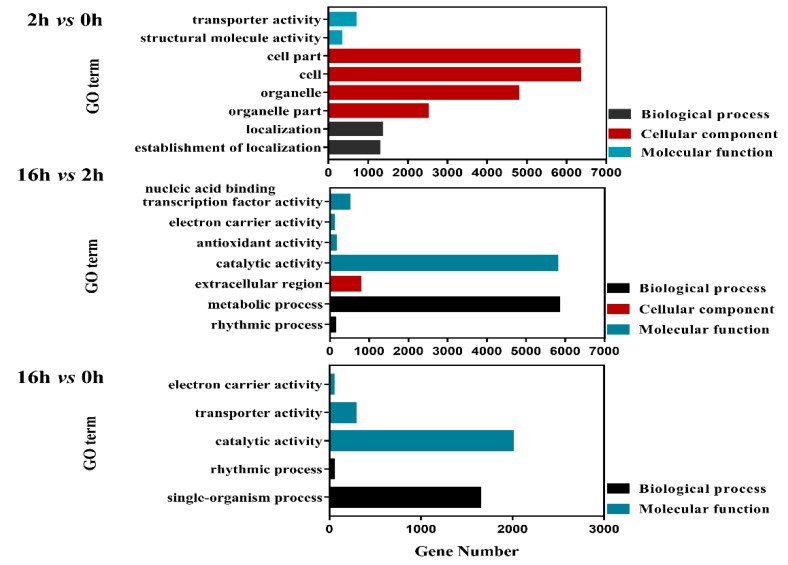
Bar plots of GO enrichment of DEGs with increased expressions (*q* value < 0.05).

**Figure 5:**
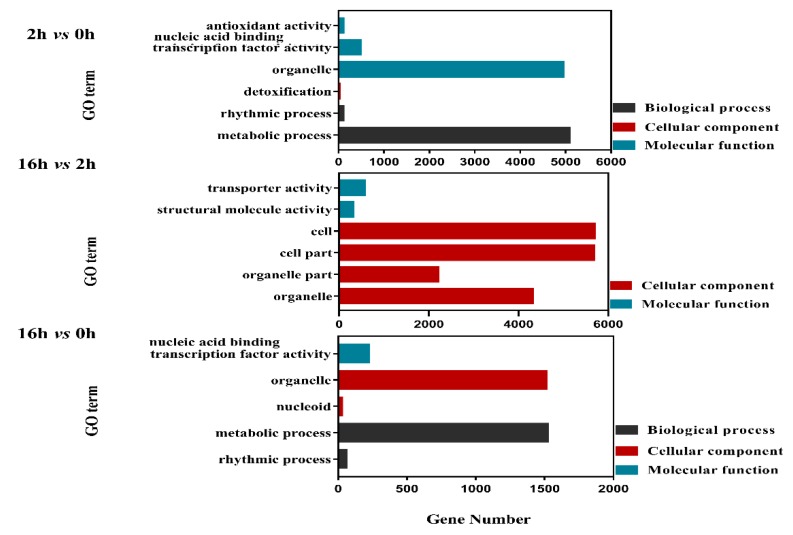
Bar plots of GO enrichment of DEGs with decreased expressions (*q* Value < 0.05).

For DEGs with increased expressions in the 2 *vs* 0 h regime, the top enriched GO terms were “Establishment of localization” and “Localization” in the category of biological process, “Structural molecule activity” and “Transporter activity” in the molecular function group. In the 16 *vs* 2 h regime, “Metabolic process” was the most highly enriched in the biological process, while “Catalytic activity” was distinctly classified in molecular function, and “Extracellular region” was clearly abundant in the cellular component category. In 16 *vs* 0 h, “Single-organism process” and “Catalytic activity” were enriched in biological process and molecular function categories, respectively (Figure 4). For DEGs with decreased expressions in 2 *vs* 0 h regime, the top enriched GO terms were “Metabolic process” and “Organelle” in the group of biological process and molecular function, respectively. In 16 *vs* 2 h, “Structural molecule activity”, “Transporter activity”, “Cell”, “Cell part” were dramatically integrated into the molecular function and cellular component category. Analogously, the “Rhythmic process”, “Metabolic process”, “Nucleoid”, “Organelle” and “Nucleic acid binding transcription factor activity” were highly enriched in 16 *vs* 0 h (Figure 5).

### 2.5. KEGG Pathway Analysis of DEGs

**Figure 6:**
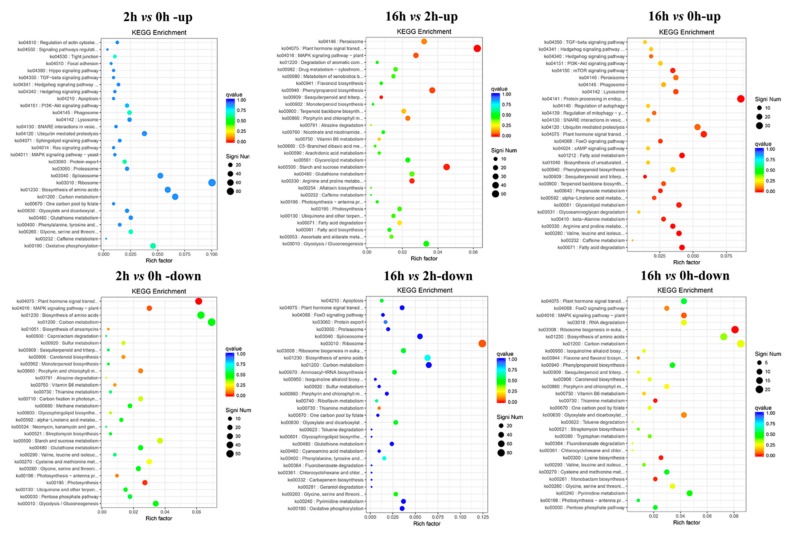
Kyoto Encyclopedia of Genes and Genomes (KEGG) enrichment of up- and down-regulated DEGs during the drought period.

All the DEGs in 2 *vs* 0 h, 16 *vs* 2 h and 16 *vs* 0 h regimes were subjected to KEGG pathway enrichment analysis. The DEGs with increased expression were enriched in “Oxidative phosphorylation” (ko00190), and “Protein export” (ko03060) pathways in 2 *vs* 0 h; in “Plant hormone signal transduction” (ko04075), “Starch and sucrose metabolism” (ko00500), “Arginine and proline metabolism” (ko00330), and “Sesquiterpenoid and triterpenoid biosynthesis” (ko00909) pathways in 16 *vs* 2 h (Figure 6); in “Fatty acid degradation” (ko00071), “Valine, leucine and isoleucine degradation” (ko00280), “Sesquiterpenoid and triterpenoid biosynthesis” (ko00909), and “Protein processing in endoplasmic reticulum” (ko04141) in 16 *vs* 0 h (Figure 6). The enriched pathways of DEGs with decreased expression were “Plant hormone signal transduction” (ko04075) and “Photosynthesis” (ko00195) in 2 *vs* 0 h, “Ribosome” (ko03010) and “Thiamine metabolism” (ko00730) in 16 *vs* 2 h, “Ribosome biogenesis in eukaryotes” (ko03008), “Lysine biosynthesis” (ko00300), and “Thiamine metabolism” (ko00730) in 16 *vs* 0 h (Figure 6). For example, a total of 74 DEGs in 2 *vs* 0 h regime, 79 DEGs in 16 *vs* 2 h, and 25 DEGs in 16 *vs* 0 h involved in “Plant hormone signal transduction” (Figure 6).

### 2.6. DEGs Related to Plant Hormone Biosynthesis and Signal Transduction

**Figure 7:**
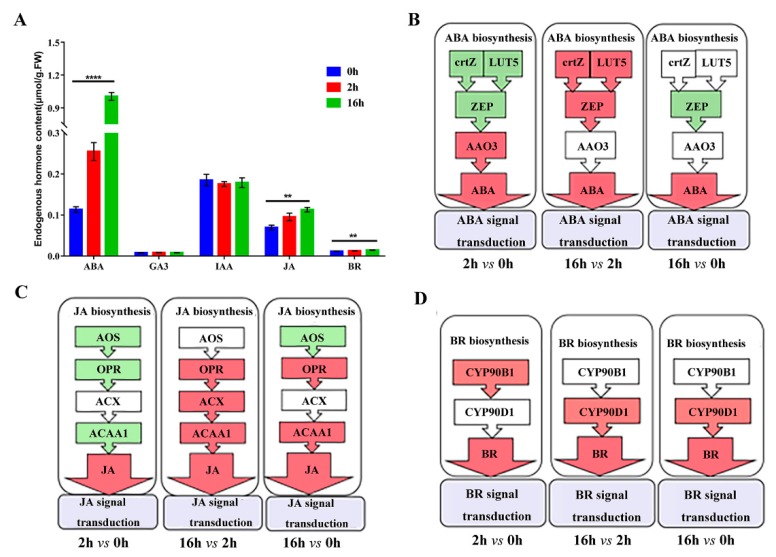
Plots showing the effects of drought stress on plant hormone synthesis in Kentucky Bluegrass. (**A**) Leaf hormone contents at 0, 2, and 16 h after drought stress, (**B**) DEGs predicted to be involved in “Abscisic acid biosynthesis”, (**C**) DEGs predicted to be involved in “Jasmonic acid biosynthesis”, (**D**) DEGs predicted to be involved in “Brassinosteroid biosynthesis”. In panel **B**, **C**, and **D**, red color represents up-regulated genes, green color represents down-regulated genes, white color represents unchanged genes.

**Figure 8:**
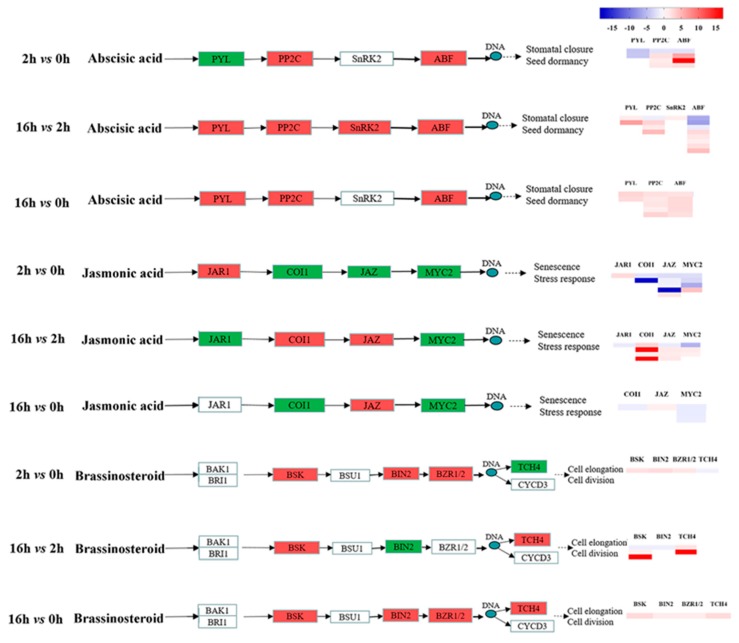
Heat maps of the expression of “Plant hormone signal transduction pathway” genes during the drought period. Red represents up-regulated genes, green represents a down-regulated gene, white represents no significant change. Red and blue represent up- and down-regulated transcripts, from the three comparisons: 2 *vs* 0 h, 16 *vs* 2 h, 16 *vs* 0 h (|log2(fold change)| > 2, *p* < 0.05).

Our work demonstrated that the four DEGs encoding beta-carotene 3-hydroxylase (*crtZ*), *beta-ring hydroxylase* (*LUT5*), *zeaxanthin epoxidase* (*ZEP*) and *abscisic-aldehyde oxidase* (*AAO3*) were differentially expressed under drought stress in ABA biosynthesis (M00372) (Figure 7B). In 2 *vs* 0 h, DEGs encoding *AAO3* were up-regulated, while those encoding *crtZ*, *LUT5*, and *ZEP* were down-regulated. In the 16 *vs* 2 h regime, DEGs encoding *crtZ*, *LUT5*, and *ZEP* were up-regulated. Genes encoding ABA receptor PYR/PYL family (*PYL*) were up-regulated in 16 *vs* 2 h and 16 *vs* 0 h regimes, and down-regulated in 2 *vs* 0 h regime (Figure 8). In 16 *vs* 2 h, *SAPK8* of SnRK2 was up-regulated. Importantly, genes encoding protein phosphatase 2C (*PP2C*) and *ABA-responsive element binding factor* (*ABF*) were up-regulated during the whole drought stress period.

Jasmonic acid biosynthesis-related genes (M00113), encoding *hydroperoxide dehydratase* (*AOS*), *12-oxophytodienoic acid reductase* (*OPR*), *acyl-CoA oxidase* (*ACX*), and *acetyl-CoA acyltransferase 1* (*ACAA1*), were differentially expressed under drought stress (Figure 7C). In 16 *vs* 2 h, DEGs encoded *OPR*, *ACX* and *ACAA1* were up-regulated. Two JA signal transduction-related genes encoding jasmonic acid-amino synthetase (*JAR1*) were significantly up-regulated after 2 h of drought stress, while the genes encoding *coronatine-insensitive protein 1* (*COI1*) and jasmonate ZIM domain-containing protein (*JAZ*) were up-regulated. *JAR1, COI1,* and *JAZ* gene expressions were down-regulated in the 16 *vs* 2 h regime (Figure 8). *MYC2* genes involved in the JA signaling pathway were also significantly down-regulated under drought treatment.

Two up-regulated DEGs were involved in brassinosteroid biosynthesis (Ko00905) (Figure 7D). The DEG encoded steroid 22-alpha-hydroxylase (*CYP90B1*, *DWF4*) was up-regulated in 2 *vs* 0 h, and those encoding 3-epi-6-deoxocathasterone 23-monooxygenase (*CYP90C1*, *ROT3*) were up-regulated in 16 *vs* 0 h and 16 *vs* 2 h regimes. BR signal transduction-related genes, encoding *BR-signaling kinase* (*BSK*), protein *brassinosteroid insensitive 2* (*BIN2*), *brassinosteroid resistant 1/2* (*BZR1* and *BZR2*) and *xyloglucosyl transferase TCH4* (*TCH4*), were significantly up-regulated in 16 *vs* 0 h regimes. *BSK* and *TCH4* genes were obviously up-regulated in the 16 *vs* 2 h regime with a log_2_(fold change) of 17.26 and 16.72, respectively (Figure 8).

### 2.7. MAPK Signaling Transduction and Interaction with Hormonal Signaling Transduction

**Figure 9:**
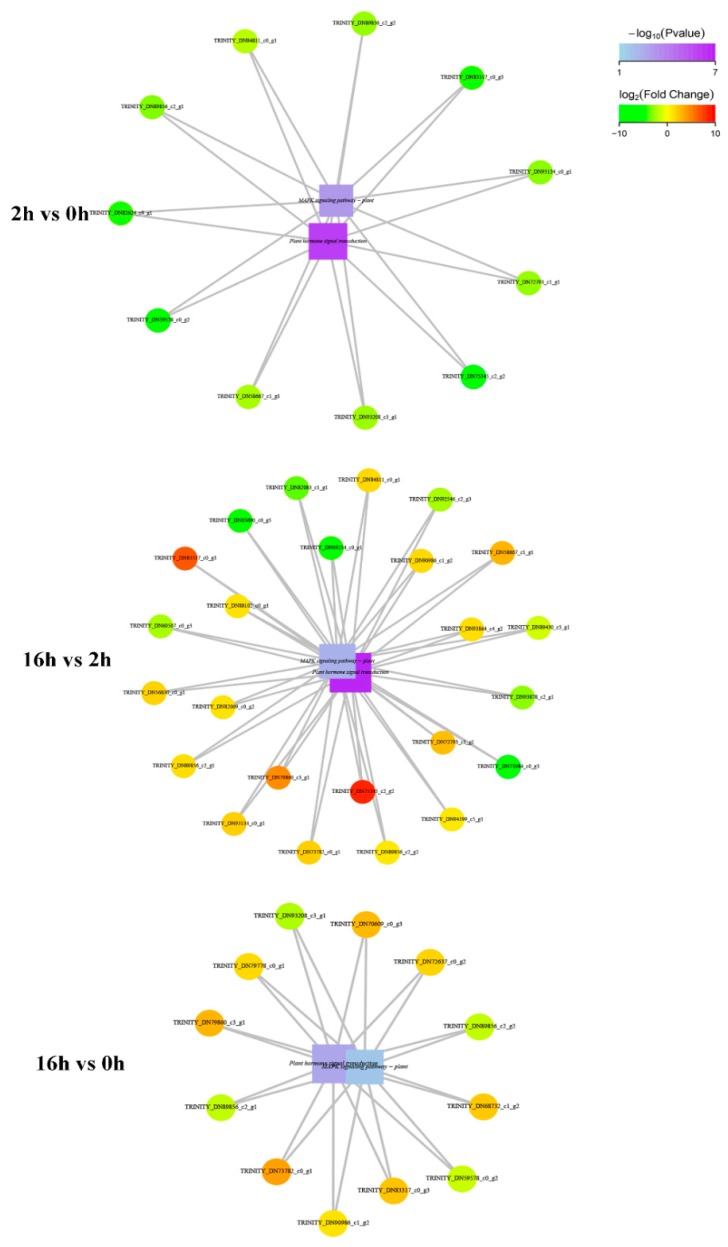
Each network diagram is composed of statistically significant functional and DEGs enrichments between “Plant hormone signal transduction” and the “MAPK signaling pathway-plant” in response to drought stress. The square represents functional information, circle indicates genes, and line represents the correlation between genes and functions. The color of the circle represents the degree of difference in gene expression, i.e., log_2_(Fold change) value, with green color representing down-regulation and red color showing up-regulation, respectively. The color of the square shows the functional enrichment, i.e., P value, where a higher enrichment may indicate a lower P value and a darker color.

We found that the “Plant hormone signal transduction” and the “MAPK signaling pathway-plant” had significant interactions. Eleven down-regulated genes were involved in networks upon the preliminary stage, including *EIN4* (TRINITY_DN75345_c2_g2) and *MYC2* (TRINITY_DN59578_c0 _g2) (Figure 9). 24 DEGs were involved in networks interactions upon the persistent drought, including *EIN4* (TRINITY_DN75345_c2_g2), *PLY8* (TRINITY_DN83317_c0_g3), pathogenesis-related protein 1-5 (TRINITY_DN73984_c0_g3) and hypothetical protein F775 (TRINITY_DN69254 _c0_g1) (Figure 9). In addition, 12 DEGs were involved in 16 *vs* 0 h, including *EIN4* (TRINITY_DN75345_c2_g2) and *MYC2* (TRINITY_ DN59578_c0_g2) (Figure 9).

### 2.8. Validation of RNA-Seq Data by qRT-PCR

**Figure 10:**
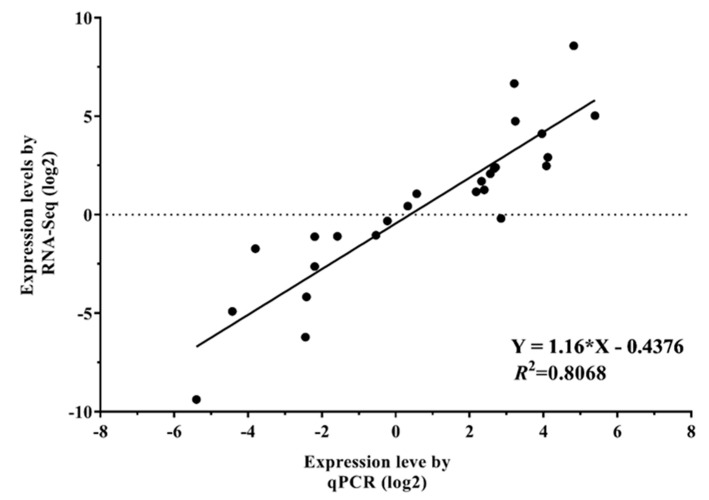
qRT-PCR validations of differentially expressed genes in a leaf of Kentucky bluegrass under drought stress. Correlations of expression level analyzed by log2 qPCR (*x*-axis) with data obtained using RNA-Seq platform (*y*-axis).

In order to confirm the results of RNA-Seq, 26 DEGs under drought stress were chosen for quantitative real-time PCR (Genes ID and primers were in Supplementary Table S5). As shown in Figure 10, the results from the qRT-PCR were highly correlated with the RNA-Seq results (*y* = 1.16 × *X*−0.4376; *R*^2^ = 0.8068; *p* Value < 0.0001), suggesting that accuracy and effectiveness of RNA-Seq results were valid for data analysis.

### 2.9. Effects of Drought Stress on Endogenous Hormones

The amount of ABA, JA and BR increased significantly with the extension of the drought period. The ABA content remarkably increased from 14.62 ng/g FW (0 h) to 67.35 ng/g FW (2 h), and finally reached 265.50 ng/g FW at 16 h. The content of JA increased by 37.26% at 2 h and 62.68% at 16 h (*p* < 0.01) after drought stress. Similar to the changing tendencies of ABA and JA, the content of BR also increased by 5.10% at 2 h and 22.16% at 16 h (*p* < 0.01) compared to the control group at 0 h (5.74 ng/g FW). GA_3_ and IAA contents did not significantly change under different drought periods (Figure 7A).

### 2.10. Transcription Factors Associated with Drought Tolerance

**Figure 11:**
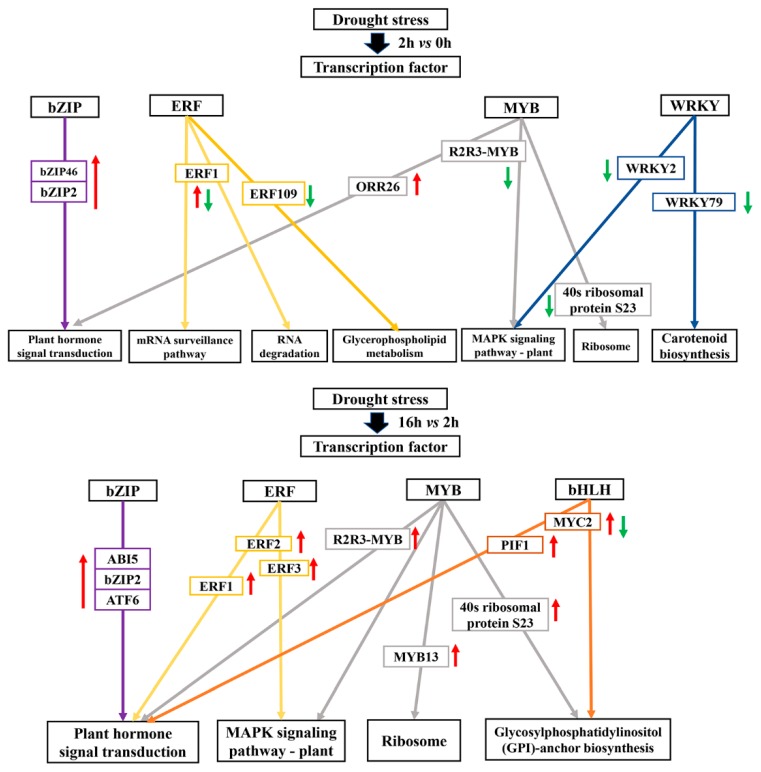
Proposed working model for drought stress transcription factor signaling network in Kentucky bluegrass. The red arrow represents the up-regulated gene, the green arrow represents the down-regulated gene. The purple frame represents the bZIP family transcription factor, the yellow frame represents the ERF family transcription factor, the gray frame represents the MYB family transcription factor, the blue frame represents the WRKY family transcription factor, and the orange frame represents the bHLH family transcription factor.

In our study, some DEGs were enriched in “transcription factor activity”. The expressions of transcription factors including *bZIP*, *bHLH*, *WRKY*, *AP2/ERF*, and *MYB* family genes significantly changed under drought stress (Figure 11). For instance, the drought-responsive genes of the bZIP family (*bZIP46*, *bZIP2*, and *ABI5*) were all up-regulated during the drought period. In addition, R2R3-MYB and ERF2 represented the key protein mediating crosstalk between “Plant hormone signal transduction” and “MAPK signaling pathway-plant” in response to drought. Furthermore, WRKY family genes were significantly induced in the early drought stage, and bHLH family genes were regulated during the extending drought period (Figure 11).

## 3. Discussion

To survive under abiotic stresses, plants often activate signaling cascades, resulting in the accumulation of endogenous hormones, and induction of defense responses [27]. Phytohormones play a key role in adaptation to the abiotic stresses by influencing growth and development. ABA is a well-documented stress-responsive hormone, while the other hormones such as JA and BRs have also been known as a key stress related hormones [28]. In the current study, contents of ABA, JA, and BRs increased during drought period, which is in accordance with previous studies [29,30].

### 3.1. Hormone Biosynthesis

Drought stress activated ABA biosynthesis and its accumulation which further aggravated plant drought tolerance, such as in tall fescue (*Festuca arundinacea*) [31]. Most of ABA biosynthetic pathway genes were up-regulated under water scarcity [32]. In the current study, most ABA biosynthetic pathway genes, except for *AAO3*, were down-regulated in the initial drought stage, whereas the ABA content was increased. This probably implied that the increase of ABA is due to the increased expression of *AAO3*. This result is consistent with the previous report on Arabidopsis (*Arabidopsis thaliana*) [33]. We recorded that PEG-induced drought stress decreased the expression of genes encoding *crtZ*, *LUT5*, and *ZEP* in the initial drought stage, but these genes increased in the extending drought period. The over-expression of *ZEP* genes confers increased drought tolerance [34]. Our results indicated that *AAO3* may play roles in ABA accumulation in the initial drought stage, while *crtZ*, *LUT5*, and *ZEP* were important in the extending drought stage.

Endogenous JA increases drought tolerance of plants [35]. In our study, the expression of JA biosynthesis genes *AOS*, *OPR*, and *ACAA1* decreased during the initial drought stage, while the expression of *OPR*, *ACX*, and *ACAA1* increased during the extending drought stage (Figure 7). The drought-tolerant variety of chickpea (*Cicer arietinum*) responded better to drought stress with earlier activation of *AOS* and *OPR* [35]. Our result is inconsistent with this, probably due to the interspecific differences. *ACAA1* belongs to multiple KEGG pathways, for instance, “Peroxisome” (ko04146). The decreased expression in the initial stage and increased expression in the extending stage of *ACAA1* probably reflects the change of H_2_O_2_ with the time course. Increased expression of *ACX* can stimulate the scavenging of H_2_O_2_ [23]. In the present study, the extending drought stress probably generated H_2_O_2_ and up-regulated the expression of *ACX*.

BRs triggered antioxidant system and increased relative water content in Arabidopsis and *Brachypodium distachyon* [36,37]. Most BR biosynthetic genes belong to the CYP90 family, such as *CYP90B1* (*DWF4*), *CYP90C1*(*ROT3*) and *CYP90D1*, catalyzing a rate-determining step in the BR biosynthetic pathways [38]. It has been reported that overexpression of *CYP90B1* in rapeseed (*Brassica napus*) rapidly increased its drought tolerance [39]. We also found *CYP90B1*and *CYP90D1* were up-regulated and may increase BR accumulation under drought stress.

### 3.2. Hormone Signaling Transduction

A change in ABA concentration leads to a signaling cascade which up-regulates genes encoding enzymes and proteins involved in drought response [40]. ABA-receptors PYL, PP2Cs, SnRK2, and ABF are pivotal players in the regulation of ABA signaling and abiotic stress responses [41]. Over-expression of *PYL2*, *PYL8*, *PP2C*, and *ABF* enhanced drought resistance of plants [42,43,44,45]. Different drought stress durations lead to diverse *PYLs*-activation to sense changes in endogenous ABA in plant [46]. In this study, *PYL8* and *PYL2* expression first decreased during the initial drought stage, and then increased in the prolonged drought stage, suggesting that these genes may have specific functions in different drought periods. Similar to our results, genes encoding PP2C4, PP2C6, and serine/threonine phosphatase 2C were highly up-regulated under drought stress in sorghum (*Sorghum bicolor*) and maize [47,48]. In this study, we found for the first time that the expression of *PP2C51* notably increased in the extending drought stage and it may play an indispensable role in drought stress tolerance. SnRK2 has a vital role in mediating adaptation to drought stress [49]. *SlSnRK2* was increased by dehydration in tomato (*Solanum lycopersicum*) leaf [50]. *SnRK2.2*, *SnRK2.3*, and *SnRK2.6* were shown to phosphorylate *ABF* and positively control the *ABF* TFs under drought stress in Arabidopsis [51]. *ABF*s are the predominant transcription factors downstream of SnRK in ABA signalling in response to drought and NaCl stress [43]. In this study, *SAPK8* of *SnRK2* and *ABF* had higher expression in the 16 *vs* 2 h regime. This result is consistent with previous studies [40]. The number of *ABFs* in the extending drought stage was more than in the initial drought stage (Figure 8), suggesting that *SnRK2* may perform crucial roles in the extending drought stage.

Expression of *JAR1* of JA signaling genes decreased conspicuously under drought stress [52]. Here, we found that *JAR1* expression was up-regulated in the early stage and decreased upon the persistent drought. In addition, the expression of *COI1* involved in stomatal movements increased after drought treatment [53]. Over-expression of *OsJAZ6* in rice resulted in advanced tolerance to mannitol stresses [54]. In this study, drought stages significantly affected the expression of *COI1s* and *JAZs*. They were inhibited in the initial stage but remarkably activated upon the prolonged drought. This result suggested that the prolonged drought tolerance may be related to the JA signaling pathway. *MYC2* is a pivotal bHLH transcription factor regulating the expression of JA responsive genes and it could be repressed by the JAZ protein [55]. It has been reported that *MYC2* transcription was prominently lower in response to dehydration stress [56]. Our results also showed that most of the DEGs encoding MYC2 family proteins had lower expression levels upon drought stress.

Members of the BSK family, such as *BSK5*, stimulated drought tolerance in Arabidopsis [57]. We identified that *BSKs* were progressively increased in drought periods, which indicated that they might be a positive regulator of drought tolerance. *BIN2* is a negative regulator of BR biosynthesis and phosphorylates the transcription factor *BZR1* [58]. In addition, it could be an interaction site with the ABA signaling pathway [59], which may play key roles in hormone crosstalk between ABA and BR. *BIN2* was up-regulated in creeping bentgrass (*Agrostis stolonifera*) upon drought stress [60]. *BZRs* were induced under drought stress [61]. In this study, expression of *BIN2* and *BZR1* increased during the initial stage, and *BIN2* probably acted as an active kinase of *BZR1*. The *TCH4* gene of Arabidopsis was up-regulated in response to drought stress [62]. Our study had similar result that *TCH4* was dramatically increased upon the prolonged drought. We proposed that *BIN2* might play a role in activating *BZR1 and BZR2* in the primary stage, while *BSK* and *TCH4* might play crucial roles in response to prolonged drought stress.

### 3.3. MAPK Signaling Transduction and Interaction with Hormones

It has been demonstrated that MAPKs are involved in plant signal transduction in response to drought [63]. Our results showed that MAPKs participated in “MAPK signaling pathway-plant”, and were also involved in “Plant hormone signal transduction”, such as the ABA and ethylene signaling pathway. *MAPKKK18* and *MAPKK3* regulated drought stress by ABA signaling pathway in Arabidopsis, and *MAPKKK18* played regulatory roles via downstream *MAPKK3* [64]. Our findings indicated that *MAPKKK18* and *MPK2* were involved in ABA signaling in the initial drought stage, and *MPK2* was activated in the extending drought period. It is possible that the increase in ABA concentration affected the MAPK signaling pathway regulation in drought stress. CTR1–AtMKK9–AtMPK3/6 mediated ethylene signaling in Arabidopsis [65]. In the present study, ETR–MKK9–MPK3-EIN3 mediated ethylene signaling in the initial drought stage, while ETR-CTR1-EIN3 mediated ethylene signaling in the extending drought period. *ETR*, *CTR1* and *EIN3* were activated in soybean under drought stress [66]; this result is similar to our findings. *MKK3* and *MPK6* were activated by JA in Arabidopsis [67]. The JA concentration did not increase during the drought period maybe because *MKK3* and *MPK6* were not activated in this research. The expressions of *MKK5* and *PR* were also increased upon the prolonged drought period, probably due to the *pathogen resistance* (*PR*) activated by the gene *MKK5* which was located upstream of the MAPK pathway. This interaction between *MKK5* and *PR* was found previously in Arabidopsis [68]. Expression of some genes involved in the “MAPK signaling pathway-plant” was inhibited in the initial drought stage, such as *MAPKKK17/18*, *MPK1/2*, *MKK9*, *MPK3*, but was increased in the extending drought period, suggesting these MAPKs played key roles in a complete hormone signaling pathway in response to drought in KB.

### 3.4. Transcription Factors Associated with Drought Tolerance

It has been documented that members of *bZIP*, *bHLH*, *WRKY*, *ERF* and *MYB* transcription factor families confer drought tolerance [69,70]. In this study, the drought-responsive genes of *bZIP46*, *bZIP2*, and *ABI5* were up-regulated during the drought period. This result is consistent with a previous study in rice [71]. The AP2/ERF superfamily is known to be involved in response to drought [72]. *OsERF109* negatively affects ethylene biosynthesis and drought tolerance in rice [73]. Similar to these reports, we found that *ERF109* was down-regulated in the initial drought stage. However, the expression levels of the ERF family were significantly different in different drought periods. “mRNA surveillance pathway”, “RNA degradation” and “Glycerophospholipid metabolism” of ERFs were mainly affected in the preliminary drought stage, and “Plant hormone signal transduction” and “MAPK signaling pathway-plant” of ERFs were highly expressed in the extending drought stage. The majority of MYB proteins belong to the R2R3-MYB, playing key roles in response to drought stress [74]. Interestingly, it has been confirmed that *OsMYB59* plays a key role in the osmotic stress response [25], which is similar to our findings. It has been documented that one *AtWRKY40* homologue and two *AtWRKY33* homologues showed a rapid and transient induced expression pattern under drought stress [75]. Our results are consistent. The information of these transcription factors will be useful for studies on molecular adaptations to drought stress in Poaceae turfgrass in the future.

## 4. Materials and Methods

### 4.1. Plant Materials and Treatment

KB (*Poa pratensis* “Midnight Ⅱ”) was used in this study. Plants were grown in plastic pots with a mix of sand and peat (1/1, *v*/*v*) in the greenhouse, with a light period of 13/11 h (day/night), relative humidity of 65 ± 5%, average light intensity of 400 µmol m^−2^ s^−1^, and temperatures of 25/15 °C (day/night). When the seedlings were four months old, they were moved to a triangular flask for hydroponics and 1/2 strength Hoagland”s solution was used to cultivate KB seedlings. After 3 weeks, 10% PEG6000 dissolved in 1/2 strength Hoagland”s solution was used to simulate drought stress. Fresh and healthy leaf samples with three biological replicates for each treatment were taken at 0 h (15:00), 2 h (17:00) and 16 h (7:00 of the second day) after drought stress.

### 4.2. RNA Extraction, cDNA Library Construction and Sequencing

RNA of fresh leaf samples at 0 h, 2 h, and 16 h after PEG6000 treatment was extracted using NEBNext^®^ Ultra™RNA Library Prep Kit for Illumina^®^ (NEB, Ipswich, MA, USA), and tested for quality with Qubit™ RNA HS Assay Kit (Invitrogen™, Carlsbad, CA, USA). Transcriptome library for sequencing was constructed according to the NEBNext mRNA Library Prep Master Mix Set for Illumina and NEBNext Multiplex Oligos for Illumina. The libraries were sequenced via Illumina HiSeq™2500.

### 4.3. De novo Assembly

In order to ensure the quality of information analysis, the original data were filtered to obtain clean data. Trinity was used to assemble the clean data via *de novo* into transcripts [76]. The ID number of the transcript was named as TRINITY_DNa_cX_gY. For the redundant transcript obtained by Trinity assembly, the longest transcript was extracted for each unigene, which was used as the reference sequence for subsequent analysis.

### 4.4. Gene Function Annotation

Unigene annotation was conducted via NCBI Blast^+^ for CDD, KOG, COG, NR, NT, PFAM, Swissprot, and TrEMBL notes. Homology was based on BLASTx comparisons to the non-redundant database (E < 10^−5^). Gene Ontology (GO) functional annotation was based on the protein annotation results of Swissprot and TrEMBL. GO mapping, annotation, and enrichment tests were conducted using BLAST2GO Pro v3.0 following default parameters [77]. GO annotation was obtained from UniProt annotation information. KAAS and KEGG automatic annotation servers were used for KEGG annotation [78].

### 4.5. Quantification of Gene Expression Levels

The gene expression level was estimated by counting the sequencing sequences (reads) located in the regions of the genome or gene exons. The reads count was not only proportional to the actual expression level of genes but also positively correlated with gene length and sequencing depth. In order to compare the estimated gene expression levels of different genes, the concept of Transcripts Per Million (TPM) was introduced [79,80]. The gene expression levels among 0 h, 2 h and 16 h were compared, and differentially expressed genes (DEGs) were identified by DESeq (*q* Value < 0.05 and |log_2_(fold change)| > 1).

### 4.6. Gene Ontology and KEGG Pathway Enrichment Analysis

GO and KEGG were employed to identify and analyze significantly enriched functional category and metabolic pathways in DEGs. The DEGs were classified by GO (http:// www.geneontology.org/) and the GO terms of the three categories (Biological Process, Cellular Component, and Molecular Function) were assessed. The enriched pathways of DEGs were statistically significantly mapped and noted via the KEGG database (*q* Value < 0.05). R’s iGraph package was used to perform functional enrichment correlation analysis [81].

### 4.7. Validation of Transcripts by Quantitative Real-Time PCR (qRT-PCR)

Twenty-six DEGs possessing potential functional roles in regulating drought tolerance of KB were selected for validation using qRT-PCR. Total RNA was extracted from leaf using the RNAprep pure plant kit (Tiangen, Beijing, China). The qRT-PCR was performed with ABI7500 in 20 μL reactions, with 2 μL of cDNA, 2 μM of each primer, and 10 μL of TB Green Premix Ex Taq II (Takara, Japan). The thermal cycling conditions were as follows: 95 °C for 30 s, 40 cycles of 5 s at 95 °C, and 30 s at 60 °C. The relative gene expression of qRT-PCR was calculated using the 2^−^^ΔΔ*C*t^ method [82]. Scattering maps were generated from the log2 (fold change) of RNA-seq and qRT-PCR results.

### 4.8. Measurement of Endogenous Hormones

The contents of endogenous hormones of the leaf samples of KB were determined using the Enzyme-Linked Immunosorbent Assay (ELISA) technique. The method of extraction and determination of phytohormones, including gibberellin (GA), indole-3-acetic acid (IAA), ABA, JA, and BR, were measured as described by Yang et al., Zhao et al., and Wang et al. [83,84,85]. Three biological replicates were used for measurement.

## 5. Conclusions

In this study, transcriptome-level changes in KB upon short- and long-term drought stress were analyzed and genes related to drought stress responses were identified. KEGG pathway enrichment analysis of DEGs indicated that drought stress changed numerous aspects of metabolism, signaling, and transportation, such as “Plant hormone signal transduction”, “MAPK signaling pathway-Plant” “Starch and sucrose metabolism”, and “Arginine and proline metabolism”. Furthermore, the transcriptomic comparison together with hormone analysis revealed that drought stress led to hormone accumulation and then resulted in plant hormone signal transduction alterations. Expressions of genes related to hormone biosynthesis and hormone signal transduction were changed. Importantly, we suggest that *SAPK8*, *BSK*, and *ABF* were positive regulators, and *MYC2* was a negative regulator in drought periods. *PYL*, *TCH4, COI1*, and *JAZ* play an important role in drought duration. Transcription factors including *bZIP*, *bHLH*, *WRKY*, *ERF* and *MYB* family genes were also significantly enriched upon drought stress. The specific DEGs encoding functional proteins, kinase and transcription factors could be valuable information for genetic manipulation to promote drought tolerance of KB in the future.

## Data Availability

The raw data of the RNA-Seq have been submitted to NCBI Sequence Read Archive (SRA) under BioProject accession PRJNA517968.

## Supplementary Materials

Supplementary materials can be found at https://www.mdpi.com/1422-0067/20/6/1289/s1. Table S1. Summary statistics of the Kentucky Bluegrass transcriptome assemblies. Table S2. Statistics of unigene and transcript length. Table S3. Unigenes annotated in different databases. Table S4. Differentially expressed genes (DEGs) in Kentucky Bluegrass under drought stress. Table S5. The primers used for qRT-PCR. Figure S1. Species distribution of all the unigene sequences. Figure S2. The repeatability of the libraries was evaluated. Figure S3. Cluster analysis of samples. Figure S4. Comparative analysis of differentially expressed genes (DEGs) in the drought period.
