# Supplementary figures and images for: Biosynthesis and Signal Transduction of ABA, JA, and BRs in Response to Drought Stress of Kentucky Bluegrass
Source: Int J Mol Sci. 2019 Mar 14;20(6):1289. doi: 10.3390/ijms20061289 (PMC6471471; doi:10.3390/ijms20061289)

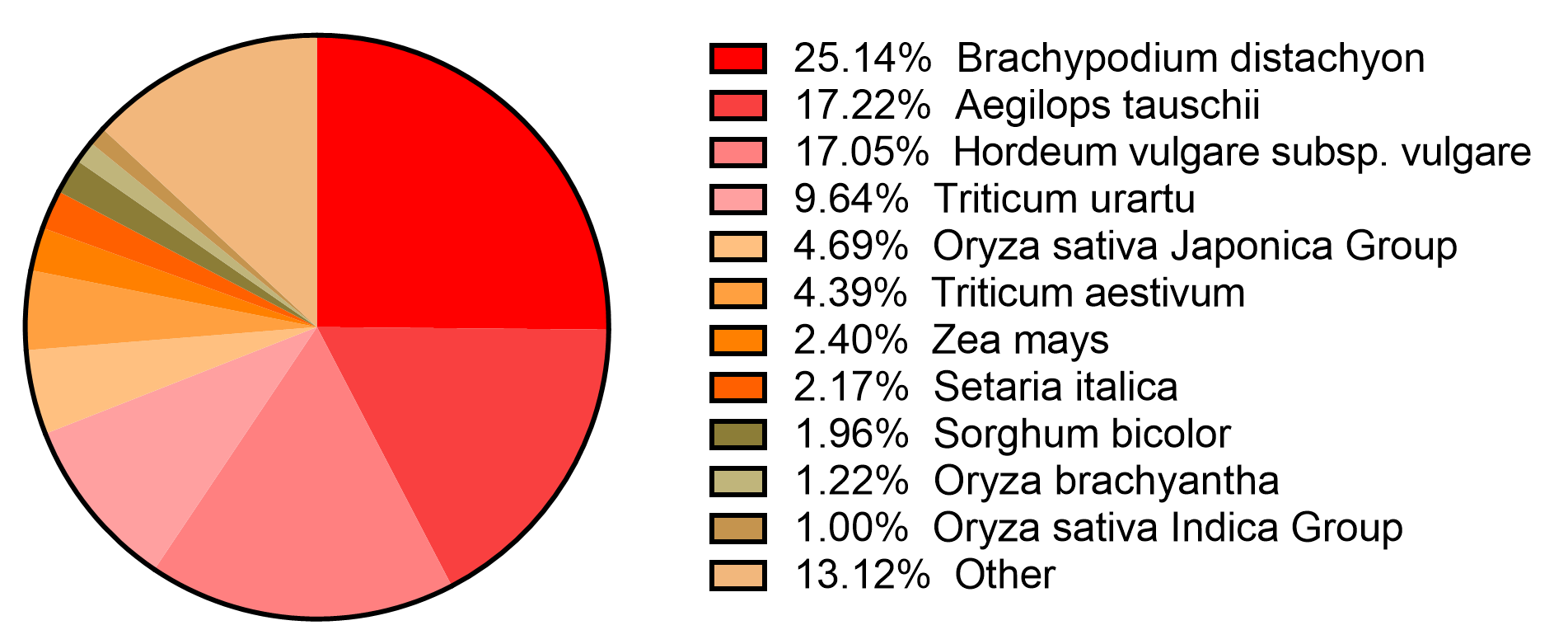

Supplement: Supplementary file 1 [file ijms-20-01289-s001.zip › Figure S1. Species distribution of all the unigene sequences.tif]

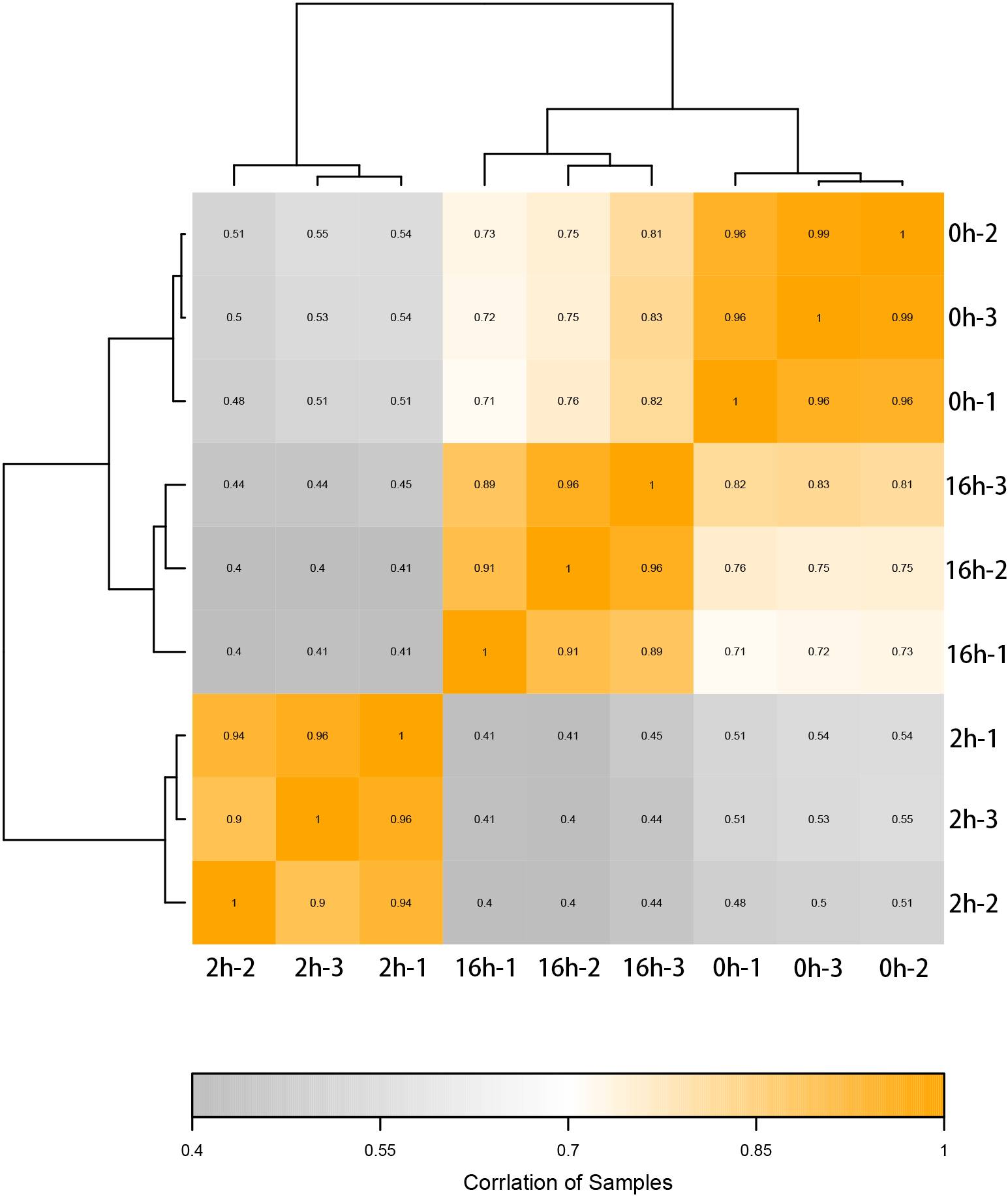

Supplement: Supplementary file 1 [file ijms-20-01289-s001.zip › Figure S2. The repeatability of the libraries wereas evaluated.tif]

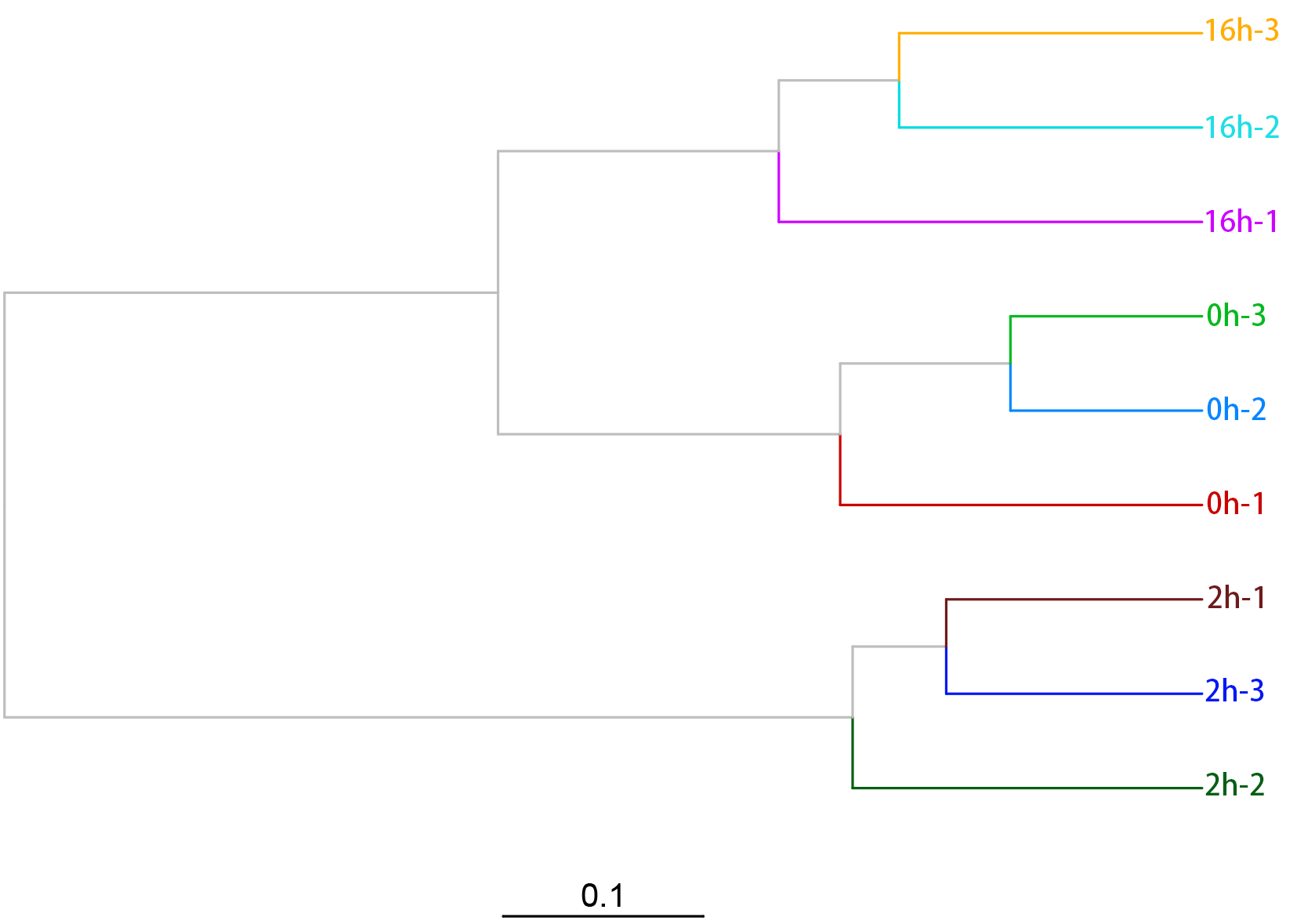

Supplement: Supplementary file 1 [file ijms-20-01289-s001.zip › Figure S3. Cluster analysis of samples.gene_tpm_bray_tree.tif]

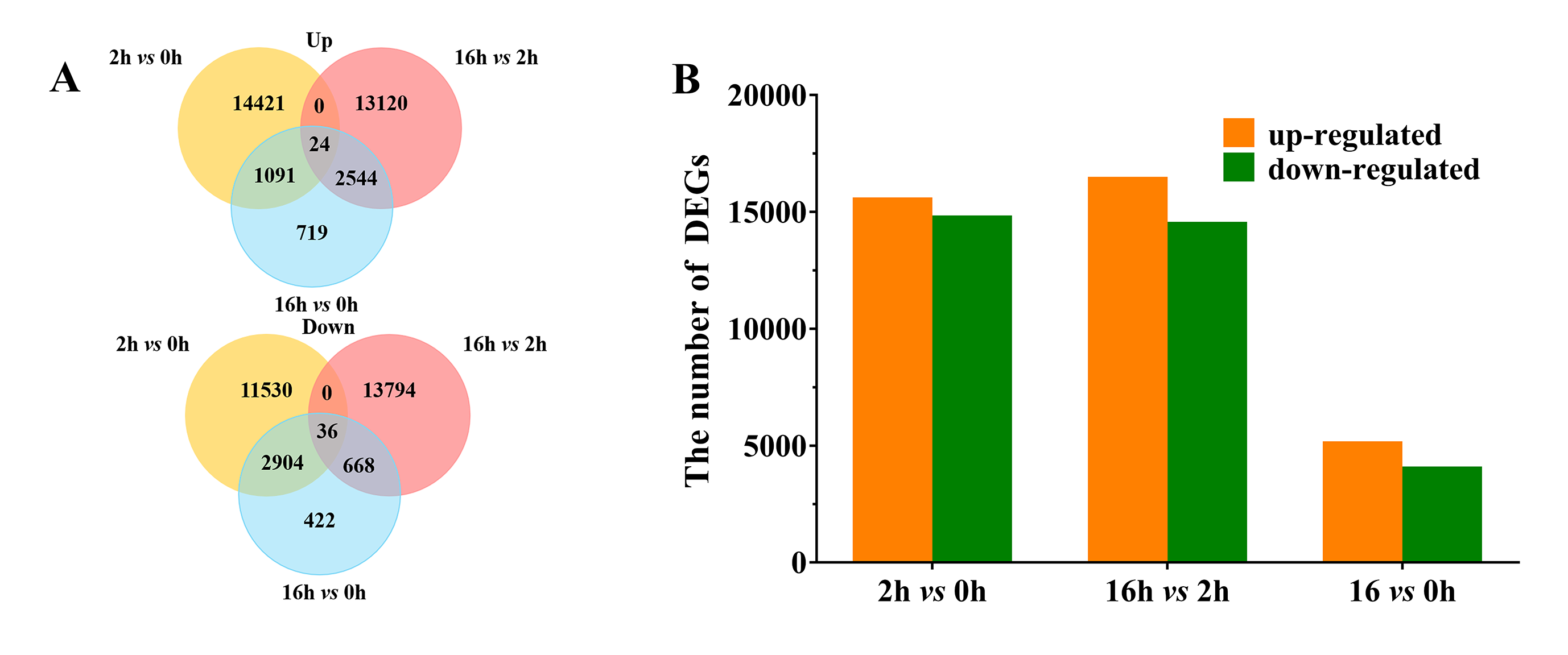

Supplement: Supplementary file 1 [file ijms-20-01289-s001.zip › Figure S4. Comparative analysis of differentially expressed genes (DEGs) in the drought period.tif]
